# Predictors of Quality of Life Six Years after Curative Colorectal Cancer Surgery: Results of the Prospective Multicenter Study

**DOI:** 10.3390/medicina58040482

**Published:** 2022-03-26

**Authors:** Gintare Valeikaite-Tauginiene, Agne Kraujelyte, Eligijus Poskus, Valdemaras Jotautas, Zilvinas Saladzinskas, Algimantas Tamelis, Paulius Lizdenis, Audrius Dulskas, Narimantas Evaldas Samalavicius, Kęstutis Strupas, Tomas Poškus

**Affiliations:** 1 Clinic of Gastroenterology, Nephrourology, and Surgery, Institute of Clinical Medicine, Faculty of Medicine, Vilnius University, Santariskiu Str. 2, 08661 Vilnius, Lithuania; eligijus.poskus@santa.lt (E.P.); valdemaras.jotautas@santa.lt (V.J.); kestutis.strupas@santa.lt (K.S.); tomas.poskus@santa.lt (T.P.); 2 Faculty of Medicine, Vilnius University, Ciurlionio Str. 21, 03101 Vilnius, Lithuania; kraujelyte.agne@gmail.com (A.K.); audrius.dulskas@nvi.lt (A.D.); 3 Clinic of Surgery, Lithuanian University of Health Sciences, Eiveniu Str. 2, 50009 Kaunas, Lithuania; zilvinas.saladzinskas@lsmuni.lt (Z.S.); algimantas.tamelis@lsmuni.lt (A.T.); paulius.lizdenis@lsmuni.lt (P.L.); 4 Clinic of Internal, Family Medicine and Oncology, Institute of Clinical Medicine, Faculty of Medicine, Vilnius University, Santariskiu Str. 2, 08661 Vilnius, Lithuania; narimantas.samalavicius@gmail.com

**Keywords:** colorectal cancer, quality of life, EORTC-QLQ-C30, EORTC-QLQ-CR29

## Abstract

*Background and objectives*: Improving early diagnosis and advances in colorectal cancer (CRC) treatment leads to longer survival of these patients. The purpose of this study was to identify the main surgical factors affecting long-term Quality of life (QoL) among colorectal cancer patients after surgery. *Materials and Methods*: QoL was prospectively evaluated in patients undergoing elective colorectal cancer resection operations in three CRC surgery centers of Lithuania using EORTC generic (QLQC-30) and disease-specific (QLQ-CR29) questionnaires at the time of preoperative admission and 1, 24, and 72 months after surgery. QoL was evaluated among different patient groups, diagnostic and treatment modalities, disease, and postoperative complications. Non-parametric tests and multivariate logistic regression models were used for statistical analysis. *Results*: Eighty-eight consecutive CRC patients from three institutions were included in the study over a three-month inclusion period, 42 (47.73%) women and 46 (52.27%) men, mean age 64.2 ± 11.5 years. Most tumors were localized in the sigmoid colon and rectum. The largest number of patients had stage III cancer. Twenty-nine patients died—a 6-year survival rate was 67%. 50 of 59 live patients (84.8%) responded to the questionnaire 6 years after their operation. Evaluating changes in quality of life 72 months after surgery with assessments before surgery, both questionnaire responses revealed good long-term CRC surgical treatment results: improved general and functional scale estimates and decreased symptom scale ratings. The multivariate analysis found that age, stoma formation, and rectal cancer were independent risk factors for having worse QoL six years after surgical intervention. *Conclusions*: Six years after surgery, QoL returns to preoperative levels. Age, stoma formation, adjuvant treatment, and rectal cancer reduce long-term QoL.

## 1. Introduction

Colorectal cancer (CRC) is one of the most burdensome cancers in the Western world due to its high incidence, significant mortality, and increasing survivorship [1,2,3]. About 1.4 million people are diagnosed with new cases of colorectal cancer worldwide every year. Colon cancer is more common in developed countries and is associated with lifestyle [4]. Improving early diagnosis and advances in treatment leads to longer survival of these patients [5,6,7]. The emergence of new treatment options for CRC, such as laparoscopic and robotic surgery, transanal techniques, and neoadjuvant and total neoadjuvant chemo- and radiotherapy, result in better outcomes, as well as increasing the quality of life of the patients. Quality of life (QoL) is a multidimensional, dynamic, subjective, and patient-centered construct comprising physical, functional, emotional, and social or family well-being [8]. Not only does it provide patient-centered outcomes of cancer treatment, but it is also related to overall survival [9,10,11] and a good indicator of treatment quality [12,13]. In addition, QoL measurements have become particularly important in assessing the outcome of long-term treatment in chronic diseases or where improvement is only short-term and temporary, and where disease progression is unstoppable and only palliative treatment is possible. Health-related quality of life (HRQoL) research is of great significance and importance, as it helps to evaluate the effectiveness of treatment methods, health improvement, and disease prevention programs, and is useful in monitoring the state of public health and developing public health policy.

Our study aims to assess the long-term results of the quality of life after surgical treatment of CRC and to determine the factors associated with decreased quality of life in the long-term postoperative period.

## 2. Patients and Methods

A prospective snapshot cohort study was performed. Lithuanian bioethics committee approval (no. L-13-03/1) was obtained. The study included 88 patients operated on with curative intent for CRC in three major cancer centers of Lithuania: Vilnius University Hospital Santaros Clinics, Lithuanian University of Health Sciences Kaunas Clinics Hospital, and the National Cancer Institute. The patients were included in the study for three months, from September to December 2012. All patients older than 18 years admitted for elective curative surgery for colorectal cancer, with the diagnosis confirmed endoscopically and histologically, were included. Patients who underwent emergency surgery were excluded. Informed consent was obtained, and baseline demographic information was collected preoperatively using patient interviews. Clinical and operative details, American Society of Anesthesiologists (ASA) grade, preoperative radiological evaluation, neoadjuvant treatment, operation type (right, left, or rectal procedure), presence of a stoma (or not), final pathological diagnosis, and postoperative complications were also recorded and reported earlier [14].

Patients’ quality of life was assessed before surgery, and at 1, 24, and 72 months after the surgery. Validated Lithuanian translations of the EORTC QLQ-C30 (version 3.0) and QLQ-CR29 questionnaires were used in the current study. The collected data were analyzed according to EORTC scoring guidelines in the same way as we reported previously [14]. The higher estimates in assessing the overall state of health and functional scales indicated better results. Higher estimates indicated more pronounced symptoms and worse postoperative outcomes. The factors that resulted in a statistically significantly worse quality of life outcome in the long-term period were identified.

Statistical analysis was performed using SPSS^®^ software version 23 (SPSS, Chicago, IL, USA). Non-parametric statistical tests and multivariate logistic regression models were used. Overall survival (OS) was calculated as the difference between the date of operation and the date of death (from any cause) or 72 months after the operation. Survival curves were estimated using the Kaplan–Meier estimator. Survival curves were compared with the log-rank test.

## 3. Results 

Eighty-eight patients were included in the study. The demographic and clinical data are shown in Table 1.

All patients were treated with surgery—58 (65.9%) patients with open-surgery, 26 (29.6%) with laparoscopic surgery, and 4 (4.5%) with transanal endoscopic microsurgery. There were no conversions in the laparoscopic group. A stoma was formed in 31 patients, with 19 (21.6%) preventive ileostomies and 12 (13.6%) end colostomies. Adjuvant chemotherapy was commenced 1-month post-operatively for 23 (26.1%) patients and radiation therapy for 5 (5.7%); in the third month, chemotherapy was used in 27 (30.68%) patients and radiotherapy in 2 (2.27%) patients.

**Figure 1:**
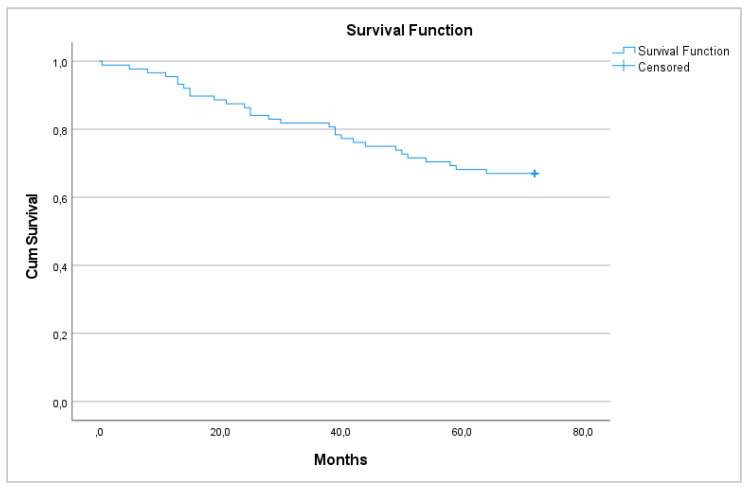
6-year overall survival.

**Figure 2:**
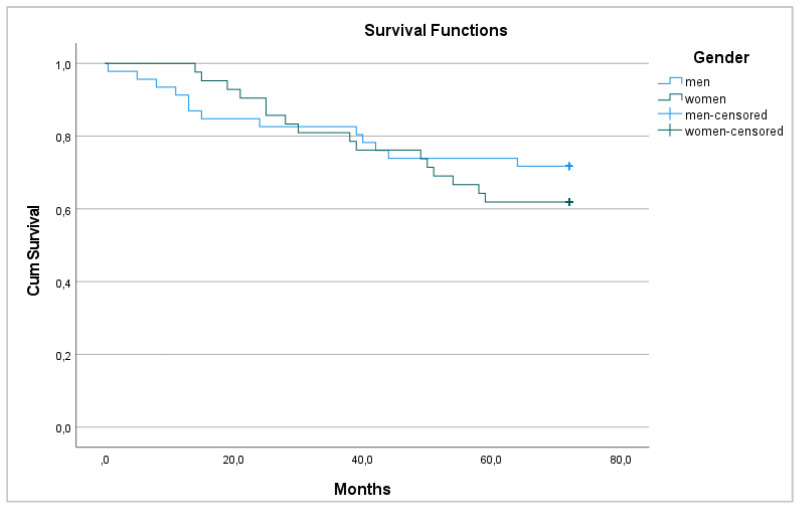
6-year overall survival between men and women.

Regarding survival, 29 patients died 72 months after surgery (33.0%), with a 6-year survival rate of 67% that was equal between men and women (*p* = 0.448) (Figure 1 and Figure 2).

**Figure 3:**
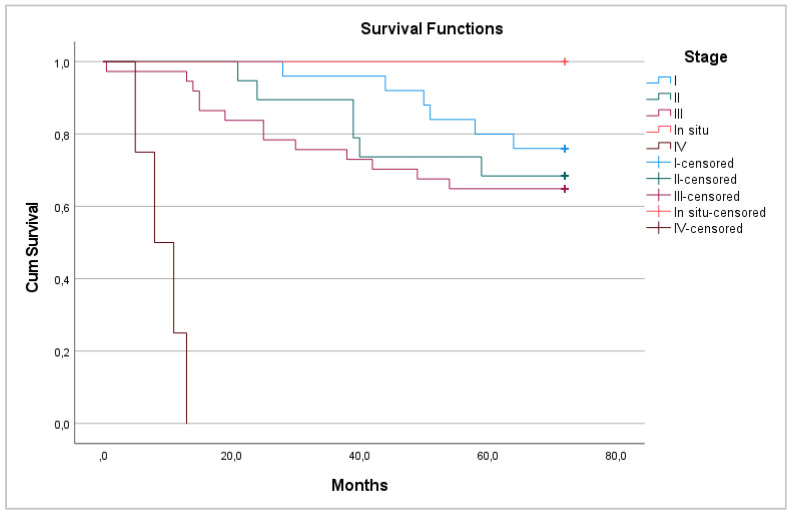
6-year overall survival between pathological stages.

Most patients had stage III cancer—37 (42%). Patients with stage IV cancer did not survive after 6 years, although all patients with a tumor in situ survived (*p* < 0.001) (Figure 3).

Fifty of the remaining 59 living patients (84.8%) responded to the questionnaire 72 months after surgery.

**Figure 4:**
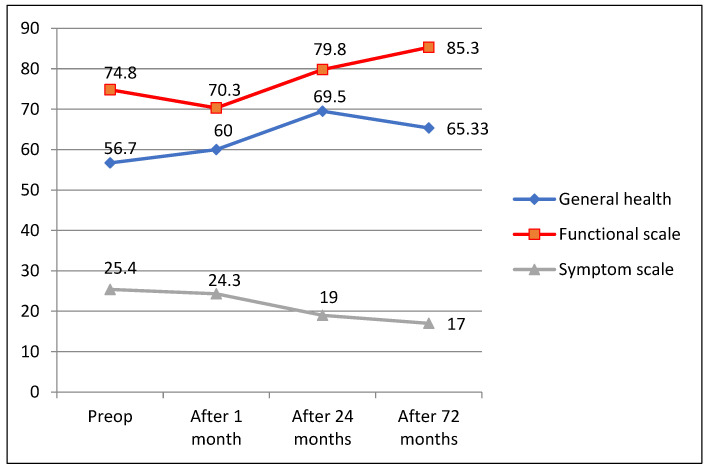
QLQ-C30 questionnaire outcomes 72 months after surgery.

**Figure 5:**
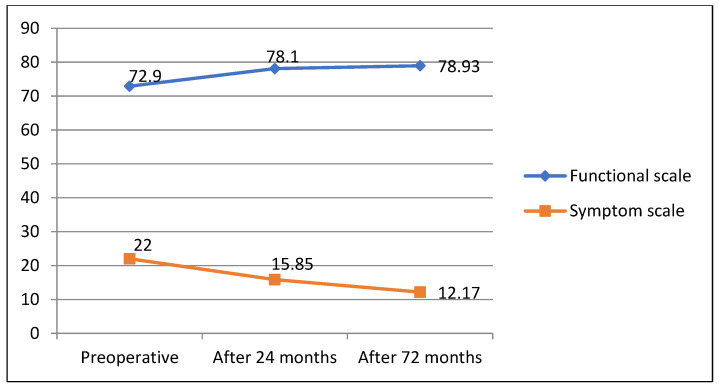
QLQ—CR29 questionnaire outcomes 72 months after surgery.

Evaluating changes in QoL 72 months after surgery with assessments before surgery both QLQ—C30 and QLQ—CR29 questionnaire responses revealed good long-term CRC surgical treatment results, showing improved general (60; 69.5; 65.33; *p* = 0.06) and functional (70.3; 79.8; 85.3; *p* = 0.041/72.9; 78.93; *p* = 0.049) scale estimates and decreased symptom scale ratings (24.3; 19; 17; *p* = 0.034)/22; 12.7; *p* = 0.025) (Figure 4 and Figure 5).

**Figure 6:**
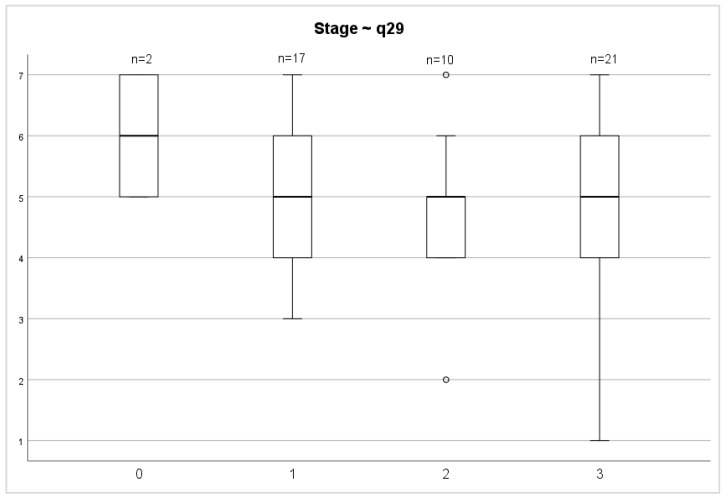
Stage and general health status.

**Figure 7:**
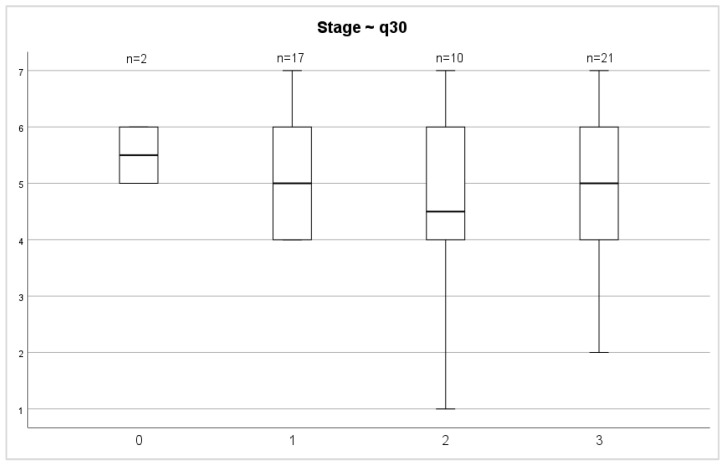
Stage and overall quality of life.

Evaluating the QoL 72 months after surgery between stages of both overall health status and overall QoL scores, we did not find any significant differences (*p* = 0.687; *p* = 0.457) (Figure 6 and Figure 7).

**Figure 8:**
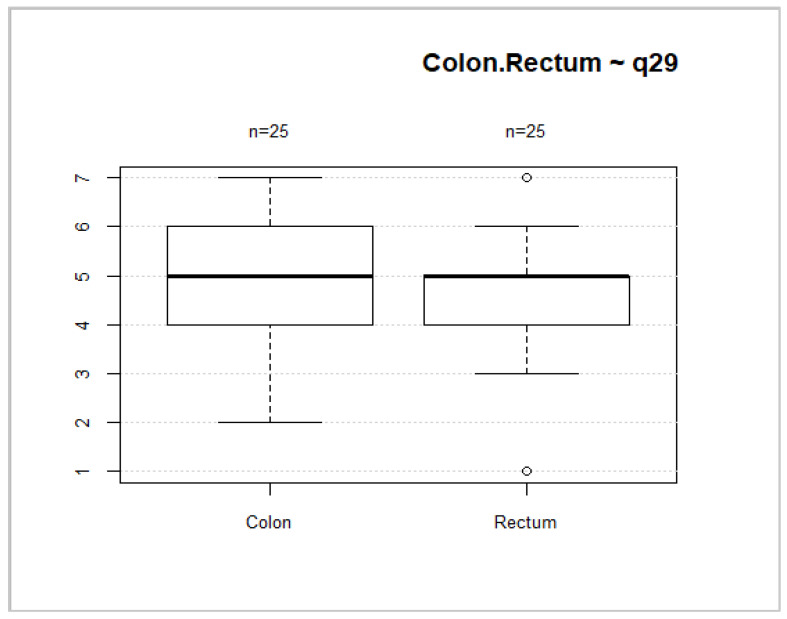
Tumor location and general health status.

**Figure 9:**
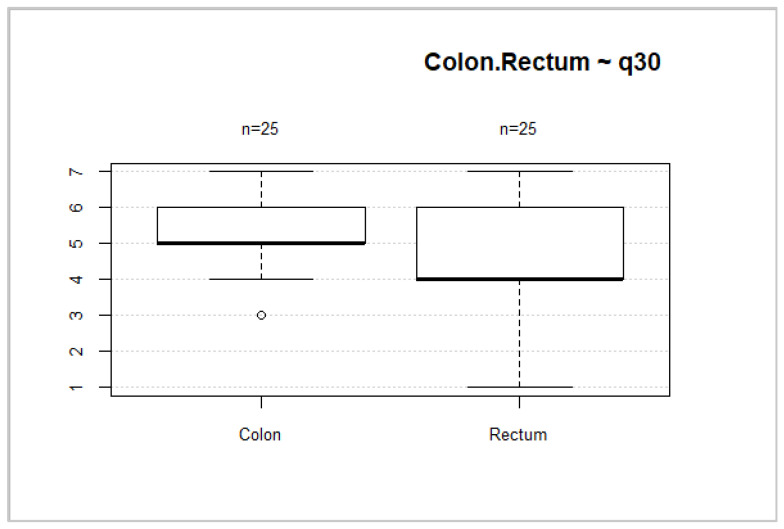
Tumor localization and overall QoL.

Forty-six (52.3%) patients had rectal cancer. This localization of tumors is associated with worse overall health status and overall QoL scores (Figure 8 and Figure 9). However, no significant differences between these groups were found (*p* = 0.2035; *p* = 0.1002).

We performed multivariate logistic regression models, which revealed that age (≥ 65 years), stoma formation, and rectal cancer are significant predictors of worse QoL (Table 2).

## 4. Discussion

We found that the QoL of patients increased two years after surgery and was maintained for up to six years. In a German study, QoL was assessed one and three years after diagnosis. Most patients with CRC reported high overall QoL and only small deficits in physical functioning, but deficits in emotional and social functioning persisted over years in patients with CRC. Improvements in QoL from the first to the third year after diagnosis in patients who remained free of disease were very modest and limited to fewer financial difficulties, a better future perspective, and fewer stoma-related problems [15].

We found that decreased long-term QoL was associated with age, stoma formation, and the use of radiotherapy. The study that investigated HRQoL in terms of symptoms and functional outcomes in disease-free survivors of rectal cancer showed that age, female sex, stoma, late complications predicted worse physical functioning; stoma and chemoradiotherapy—worse body image and age, female sex, and late major complications worse sexual functioning [16]. Another study revealed that patients with ostomies who had any late complications had lower overall HRQoL (OR 1.5; 95% CI 0.9–2.6). This was not the case for patients with anastomoses (OR 0.9; 95% CI 0.5–1.5) [17]. 

Comparing laparoscopic vs. open surgery 18 months after surgery, any differences in QoL between patients randomized to laparoscopic-assisted colectomy (LAC) or open colectomy favored LAC. However, the magnitude of the benefits was small; only age and activity were predictive of poor QoL [18]. We also didn’t find any influence of the type of surgery on QoL. The COLORII trial revealed that HRQoL after rectal cancer surgery was not affected by the surgical approach [19]. Other studies have shown the opposite. Elderly patients undergoing laparoscopic colectomy for cancer experience fewer postoperative local complications than elderly patients undergoing an open colectomy [20]. Nevertheless, in the first postoperative month, these patients experienced worse global QoL than younger patients undergoing the same operation with impairment of all functions and the presence of fatigue, sleep disturbances, appetite loss, and dyspnea [21]. HRQoL generally improved over the first year after laparoscopic colectomy, reaching even better levels than before surgery. There was an early postoperative improvement in the patients’ emotional status [22].

A prospective survey of a population-based sample of 763 colorectal cancer patients assessed sociodemographic variables, health behaviors, optimism, threat appraisal, and perceived social support at 5 months post-diagnosis as predictors of QoL and psychological distress 5 years post-diagnosis. Risk factors for worse QoL and/or greater psychological distress included later-stage disease, having a permanent stoma, rectal cancer, fatigue, smoking, being single, low social support, low optimism, and a more negative cancer threat appraisal [23].

The association between a post-diagnosis lifestyle score and HRQoL in the long-term CRC survivals indicated that lifestyle behaviors, such as a body mass index (BMI) < 30 kg/m^2^, dietary intake, physical activity, and smoking status, were associated with HRQol among CRC long-term survivors in a cross-sectional study [24].

The six-year OS of patients in our cohort (67%) was similar to those found in hospital-based studies in Brazil (63.5%) [25], Italy (66.45%) [26], and Taiwan (68.7%) [27]. We did not identify differences based on gender or tumor location, but found differences between clinical stages in OS. Similar findings were obtained in a Brazilian study [25].

The main disadvantage of this study is its small cohort. We tried to capture a snapshot of short-term surgical practice in the country, providing data on the long-term survival and QoL of CRC patients. We also included only patients undergoing elective surgery with curative intent. Emergency surgery for colorectal cancer does not influence overall and disease-free survival [28]; the data on the QoL of this small sub-group of patients should be studied further. 

## 5. Conclusions

In conclusion, most patients return to a stable QoL within 24 months after the operation, and it remains stable by 72 months. Older age, stoma formation, adjuvant treatment, and rectal cancer influence long-term quality of life. Lifestyle adjustments and social support are important in improving quality of life and should be further investigated.

## Figures and Tables

**Table 1:** Demographic and clinical data.

Characteristics	
Age (years) [mean± SD]:	64.2 ± 11.5
Gender [*n* (%)]:	
Men	46 (52.27%)
Women	42 (47.73%)
ASA grade [*n* (%)]:	
I	8 (9.09%)
II	38 (43.18%)
III	40 (45.46%)
IV	2 (2.27%)
Tumor location [*n* (%)]:	
Colon	42 (47.73%)
Rectum	46 (52.27%)
Neoadjuvant treatment [*n* (%)]:	
Radiotherapy:	14 (15.90%):
Short course radiotherapy	2 (2.27%)
Long course radiotherapy	12 (13.64%)
Chemotherapy (in addition)	11 (12.50%)
Type of operation [*n* (%)]:	
Open	58 (65.91%)
Laparoscopic	30 (34.09%)
Stage [*n* (%)]:	
In situ	3 (3.41%)
I	25 (28.41%)
II	19 (21.59%)
III	37 (42.05%)
IV	4 (4.54%)
Hospital stay (days) [mean ± SD]:	9.9 ± 4.0

**Table 2:** Multivariate analysis of factors predicting poorer quality of life.

	Factor	Value	Std. Error	T Value	*p*-Value	OR
	Gender (men)	−0.738	0.886	−0.834	0.405	0.478
	Age (≥65 years)	−0.122	0.043	−2.834	0.005	1.130
Global health	Tumor location (rectum)	4.109	2.131	1.928	0.054	60.887
status/QoL	Neoadjuvant treatment	−3.276	2.017	−1.624	0.104	0.038
	Stage (III)	−2.123	1.906	−1.114	0.265	0.120
	Stoma	2.831	1.637	1.729	0.084	16.955
	Adjuvant treatment	0.136	0.972	0.140	0.889	1.146
	ASA (III–IV)	2.249	1.343	1.674	0.094	9.478
	Operation type (open)	−1.456	1.022	−1.424	0.155	0.233
	Gender (men)	−0.3194	0.3895	−0.82	0.412	0.727
	Age (≥65 years)	−0.0301	0.0158	−1.90	0.050	1.031
Functional scale	Tumor location (rectum)	0.4095	0.9399	0.44	0.663	1.506
	Neoadjuvant treatment	1.5647	1.6295	0.96	0.337	4.781
	Stage (III)	−0.5621	0.8546	−0.66	0.511	0.570
	Stoma	−4.044	2.082	−1.943	0.050	57.043
	Adjuvant treatment	−3.987	1.676	−2.379	0.017	0.019
	ASA (III–IV)	−0.1952	0.5530	−0.35	0.724	0.823
	Operation type (open)	−0.0825	0.4866	−0.17	0.865	0.921
	Gender (men)	−1.412	1.014	−1.393	0.164	0.244
	Age (≥65 years)	−0.170	0.055	−3.108	0.002	1.185
Symptom scale	Tumor location (rectum)	−3.512	1.802	−1.949	0.050	2.049
	Neoadjuvant treatment	0.800	1.656	0.483	0.63	2.226
	Stage (III)	0.035	1.112	0.031	0.98	1.035
	Stoma	−5.877	2.592	−2.267	0.023	36.806
	Adjuvant treatment	−0.093	1.242	−0.075	0.940	0.911
	ASA (III–IV)	−0.243	1.400	−0.174	0.862	0.784
	Operation type (open)	−1.707	1.194	−1.429	0.153	0.181

## Data Availability

The data presented in this study are available on request from the corresponding author. The data are not publicly available due to institutional restrictions.
